# CCR3 Is Associated with the Death of a Photoreceptor Cell-line Induced by Light Exposure

**DOI:** 10.3389/fphar.2017.00207

**Published:** 2017-04-18

**Authors:** Yoshiki Kuse, Kazuhiro Tsuruma, Yusuke Kanno, Masamitsu Shimazawa, Hideaki Hara

**Affiliations:** Molecular Pharmacology, Department of Biofunctional Evaluation, Gifu Pharmaceutical UniversityGifu, Japan

**Keywords:** CCR3, retinal photoreceptors, light damage, *in vitro* model, retina

## Abstract

The C-C chemokine receptor type 3 (CCR3) is the receptor for eotaxins (CCL-11, 24, 26), RANTES (CCL-5) and MCP-3 (CCL-7). It was reported that an inhibition of CCR3 by antagonists or antibodies reduces the degree of laser-induced choroidal neovascularization in mice, a model for wet age-related macular degeneration (AMD). Although several chemokine receptors have the potential of reducing the degree of the chronic inflammation in experimental dry AMD, the association of CCR3 remains unknown. The purpose of this study was to determine the role played by CCR3 in the death of 661W cells which are cells of a murine photoreceptor-derived cell line as an *in vitro* model of dry AMD. The expression of CCR3 was increased in the 661W cells after light exposure. Inhibition of CCR3 reduced the rate of cell death induced by light exposure. A blockade of CCR3 signaling by CCR3 silencing and two kinds of CCR3 antagonists, SB 328437 and SB 297006, reduced the rate of light-induced cell death. In addition, CCR3 inhibition decreased the level of reactive oxygen species and the activation of caspase-3/7 induced by light exposure. These findings indicated that the CCR3 blockade should be considered for the treatment of the dry AMD.

## Introduction

There are two types of age-related macular degeneration (AMD), atrophic (dry) and exudative (wet) AMD. AMD is an important retinal disease because it causes a severe decrease of central vision, and it affects more than 10 million people in the world. The reduction of vision in wet AMD is caused mainly by choroidal neovascularization (CNV) and wet AMD patients make-up 10 to 20% of all AMD patients (Ambati et al., 2003). The pathological processes in eyes with dry AMD induce retinal pigment epithelium (RPE) atrophy (Liang and Godley, 2003) and enhance the degree of photoreceptor cell death caused by excessive light exposure and oxidative stress (Shahinfar et al., 1991; Beatty et al., 2000). The photoreceptor cell death that is accelerated by oxidative stress leads to a generation of reactive oxygen species (ROS) (Dunaief et al., 2002). The damage of the photoreceptors and RPE cells in the macula is the cause of the reduced vision, and geographic atrophy of the fundus in eyes with dry AMD.

Anti-vascular endothelial growth factor (VEGF) therapy is the most commonly used treatment of eyes with wet AMD, but there is currently no effective therapy for dry AMD patients. It was recently reported that the C-C chemokine receptor type 3 (CCR3) is expressed in CD31-positive endothelial cells in the CNV membranes of eyes with wet AMD (Takeda et al., 2009). They also showed that an inhibition of CCR3 reduced the degree of laser-induced CNV in mice more strongly than anti-VEGF antibody treatment. This finding suggested that CCR3 may be a target for the treatment for wet AMD. However, the association of CCR3 to the pathology of dry AMD has not been examined in any detail.

It is thought that ROS is closely associated with the development of the pathology of dry AMD as mentioned above. An earlier study suggested that ROS increased the expression of CCR3 in choroidal endothelial cells (Wang et al., 2011). However, it remains unknown how ROS affects the CCR3 signaling in retinal neurons such as retinal photoreceptor cells.

It has been reported that murine photoreceptor-derived cell line 661W cells are light-sensitive and caused the photo-oxidative stress (Krishnamoorthy et al., 1999; Yang et al., 2007). The purpose of this study was to determine whether an inhibition of CCR3 will affect the *in vitro* light-induced damage of the cells of a photoreceptor cell line which has been used as a model of dry AMD.

## Materials and Methods

### Cell Cultures

The studies were performed on 661W cells, an immortalized retinal cell line derived from mouse retinal tumors that were provided by Dr. Muayyad R. Al-Ubaidi (University of Houston, Houston, TX, USA). The 661W cells express SV40 T antigen, blue and green cone pigments, transducin, and cone arrestin, but not rod-specific antigens, such as opsin and arrestin or rod- and cone-specific proteins. The cells were incubated in Dulbecco’s modified Eagle medium (DMEM; Nacalai Tesque Inc, Kyoto, Japan) supplemented with 10% fetal bovine serum (FBS; Thermo Fisher Scientific, Rockford, IL, USA), 100 U/mL penicillin (Meiji Seika Kaisha Ltd., Tokyo, Japan), and 100 μg/mL streptomycin (Meiji Seika) under a humidified atmosphere of 5% CO_2_ at 37°C. The cells were released by trypsinization and passaged every 2 days.

### Western Blot Analysis

For the western blot analysis, 661W cells were seeded at a density of 3 × 10^4^ cells/well in 12-well plates, and then incubated for 24 h under a humidified atmosphere of 5% CO_2_ at 37°C. The medium was changed to DMEM with 1%FBS, and the cells were exposed to 2,500 lux of light. After 12 h, they were washed with PBS, lysed in RIPA buffer (Sigma–Aldrich, St. Louis, MO, USA) containing 1% protease inhibitor cocktail and 1% of the phosphatase inhibitor cocktails 2 and 3 (Sigma–Aldrich), and harvested. The lysates were centrifuged at 12,000 *g* for 15 min at 4°C. The protein concentration was measured with a BCA Protein Assay Kit (Thermo Fisher Scientific) with bovine serum albumin as a standard. An equal volume of protein sample and sample buffer was mixed, and the samples were boiled for 5 min at 100°C. The protein samples were separated by 5–20% SDS-PAGE gradient electrophoresis and then transferred to polyvinylidene difluoride membranes (Immobilon-P; Millipore, Bedford, MA, USA). The primary antibodies used for immunoblotting were rabbit anti-CCR3 monoclonal antibody (ab32512, Abcam, Cambridge, MA, USA), rabbit anti-phospho-nuclear factor-kappa B (NF-κB) (#3033, Cell Signaling Technology, Danvers, MA, USA), rabbit anti-NF-κB (#8242, Cell Signaling Technology) and mouse anti-β-actin mouse monoclonal (A2228, Sigma–Aldrich) antibody. A horseradish peroxidase (HRP)-conjugated goat anti-rabbit antibody (#32460, Thermo Fisher Scientific) and an HRP-conjugated goat anti mouse antibody (#32430, Thermo Fisher Scientific) were used as secondary antibodies. Immunoreactive bands were made visible by Immunostar-LD (Wako) and a LAS-4000 luminescent image analyzer (Fuji Film Co., Ltd., Tokyo, Japan). β-actin was used as the loading control.

### RNA Isolation and Reverse Transcription Polymerase Chain Reaction (RT-PCR)

661W cells were seeded at a density of 6 × 10^4^ cells/well in 6-well plates, and incubated for 24 h under a humidified atmosphere of 5% CO_2_ at 37°C. The medium was changed to DMEM with 1% FBS. The cells were exposed to 2,500 lux of light for 6 h. The RNA was extracted with NucleoSpin^®^ RNA II [Takara Bio Inc. (Kusatsu, Japan)], and single-stranded cDNA was synthesized from the total RNA with the PrimeScript^TM^ RT reagent Kit. The results are expressed as values relative to the level of *Gapdh* which was used as the internal control. The primers used for amplification were *Ccr3*: 5′-TCA ACT TGG CAA TTT CTG ACC T-3′ and 5′-CAG CAT GGA CGA TAG CCA GG-3′; and *Ccl11*: 5′-CAG ATG CAC CCT GAA AGC CAT A-3′ and 5′-TGC TTT GTG GCA TCC TGG AC-3′; and *Gapdh*: 5′-TGT GTC CGT CGT GGA TCT GA-3′ and 5′-TTG CTG TTG AAG TCG CAG GAG-3′.

Polymerase chain reaction was performed using Blend Taq^®^ Plus with a thermal cycle program of 5 min at 94°C, and 3 PCR steps of 30 s at 94°C, 30 s at 55°C and 30 s at 72°C, and finally 7 min at 72°C for a total of 45 cycles. The PCR product was transferred to 2% agarose gel for electrophoresis. The DNA bands were stained with ethidium bromide and quantified by a LAS-4000 luminescent image analyzer (Fuji Film Co., Ltd.).

### Preparation of Small Interfering RNAs

The following small interfering RNA (siRNA) sequences specific to murine *Ccr3* were used:

**Table:** 

siRNA #1:	5′-CCU GGC CUU GUA CAG CGA GAU CUU U-3′ (sense),
	5′-AAA GAU CUC GCU GUA CAA GGC CAG G-3′ (antisense),
siRNA #2:	5′-GCU UUG AGA CCA CAC CCU AUG AAU A-3′ (sense),
	5′-UAU UCA UAG GGU GUG GUC UCA AAG C-3′ (antisense).

Stealth RNAi Negative Control Medium GC Duplex #2 was used as a control. All of the siRNAs were synthesized by Thermo Fisher Scientific (Invitrogen^TM^).

The 661W cells were transfected with 50 nM of the siRNA using Lipofectamine^TM^ RNAiMAX Reagent (Thermo Fisher Scientific) and Opti-MEM (Thermo Fisher Scientific) according to the manufacturer’s protocol. After 24 h of exposure, PCR was performed to determine the degree of transfection. The effect of the two siRNAs in protecting the 661W cells from light exposure was determined.

### Light-induced Cell Death Assay in Cell Cultures

661W cells were seeded at a density of 3 × 10^3^ cells/well into 96-well plates, and incubated for 24 h under a humidified atmosphere of 5% CO_2_ at 37°C. The medium was changed, and the cells were exposed to the two CCR3 antagonists, SB 328437 [*N*-(1-Naphthalenylcarbonyl)-4-nitro-L-phenylalanine methyl ester] (Abcam), and SB 297006 (*N*-Benzoyl-4-nitro-L-phenylalanine ethyl ester) (Abcam), *N*-acetylcystein (NAC) (Wako, Osaka, Japan), or to their vehicle separately. Then, the cells were exposed to 2500 lux of white fluorescent light that the wavelength peak is 403, 435, 546, and 577 nm (Nikon, Tokyo, Japan) for 24 h.

To transfect *Ccr3* siRNA, 661W cells were seeded at a density of 1 × 10^3^ cells/well into 96-well plates without penicillin and streptomycin, and incubated for 24 h. The cells were then transfected with the negative control siRNA or *Ccr3* siRNA. After 24 h, the cells were exposed to 2500 lux of visible light for 24 h.

### Cell Death Analysis

After the light exposure, the cell death rate was calculated by double staining with two fluorescent dyes: Hoechst 33342 (Thermo Fisher Scientific) and propidium iodide (PI; Thermo Fisher Scientific). Hoechst 33342 stains the nuclei of all cells, whereas PI stains only dead cells. To determine the cell death rate, Hoechst 33342 and PI were added to the culture medium for 15 min at final concentrations of 8.1 and 1.5 μM, respectively, at the end of the culture period. Images were recorded with an Olympus IX70 inverted epifluorescence microscope (Olympus, Tokyo, Japan). The total number of cells was counted in a masked way (Y. K.) and the percentage of PI-positive cells was calculated.

For assay of apoptosis, TACS Annexin V FITC Apoptosis Detection kit (Trevigen Inc., Gaithersburg, MD, USA) are used according to manufacturer’s protocol. Briefly, Annexin V FITC (1:10 dilution), PI (1:1 dilution) and Hoechst 33342 (Invitrogen) (1:100 dilution) was diluted in 10x Binding Buffer. The buffer was added into the each wells and cells were incubated for 15 min. Images were recorded with KEYENCE Fluorescence Microscope BZ-X700 (KEYENCE, Osaka, Japan). The total number of cells was counted in a masked way (Y. K.) and the percentage of Annexin V-positive cells was calculated. We counted the nuclei of 300 cells (Hoechst-positive cells) at least for each sample in cell death analysis.

### Caspase-3/7 Activation Assay

After 24 h of light exposure, the level of caspase-3/7 in the 661W cells was determined by the Caspase-Glo 3/7 Assay (Promega, Madison, WI, USA) according to the manufacturer’s protocol. After the light exposure, caspase-Glo 3/7 reagent was added to the sample at 1:1 ratio, and the cells were incubated for 1 h at 37°C. The luminescence of each sample was measured with a microplate reader (Varioskan Flash 2.4; Thermo Fisher Scientific).

### Measurements of Cellular Reactive Oxygen Species (ROS) Production

After light exposure, 10 μM of 5-(and-6)-chloromethyl-2′, 7′-dichlorodihydrofluorescein diacetate and, acetyl ester (CM-H2DCFDA; Thermo Fisher Scientific), a free radical probe, was added to the cell cultures and incubated for 1 h at 37°C. The radical probe was converted to 2′, 7′-dichlorodihydrofluorescein (DCFH) by the intracellular esterase, and the intracellular DCFH (non-fluorescent) was oxidized to 2′, 7′-dichlorfluorescein (DCF, fluorescent) by intracellular ROS. The intensity of the fluorescence was measured by a Varioskan Flash 2.4 microplate reader (Thermo Fisher Scientific) at 485 nm (excitation) and 535 nm (emission).

### Statistical Analysis

The data are presented as the means ± standard error of the means (SEM). Student’s *t*-test or one-way ANOVA followed by Bonferroni’s test, Dunnett’s test and Tukey’s test [STAT VIEW version 5.0 (SAS Institute, Cary, NC, USA)] were used to determine the significance of any differences. A *P* < 0.05 was considered to be statistically significant.

## Results

### CCR3 Is Expressed in Cells of Photoreceptor Cell-line in Culture and Its Level Increases after Light Exposure

**FIGURE 1:**
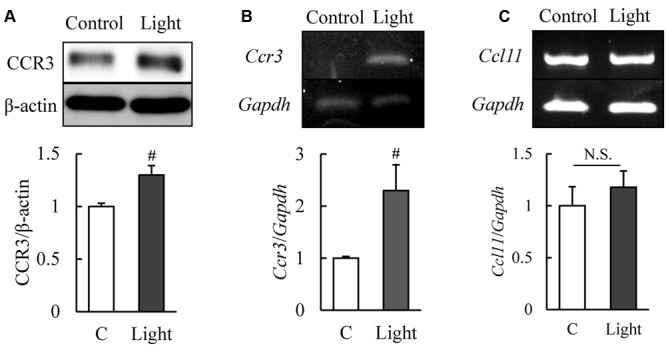
**Expression of C-C chemokine receptor type 3 (CCR3) is increased after light exposure in 661W cells, which are cells of a murine photoreceptor cell line. (A)** Level of expression of CCR3 in the 661W cells determined by western blotting. CCR3 expression is increased at 12 h after light exposure relative to the controls. **(B,C)** The level of the mRNA of *Ccr3* is increased at 6 h after light exposure without the increase of *Ccl11* which is the ligand of CCR3. Data are expressed as mean ± SEM (*n* = 6 or 7). ^#^indicates *P* < 0.05 vs. Control (C) (Student’s *t*-test).

C-C chemokine receptor type 3 is expressed in normal murine retinas (Wang et al., 2016) and our western blotting results showed that CCR3 was expressed in 661W cells, a mouse photoreceptor-derived cell line. The expression of CCR3 protein was increased significantly at 12 h after light exposure (**Figure 1A**). Moreover, the level of the mRNA of *Ccr3* was increased at 6 h after light exposure without the increase of *Ccl11* which is the ligand of CCR3 (**Figures 1B,C**). These results suggest that CCR3 signaling may be activated by light exposure although the ligand was not changed.

### CCR3 Inhibition Reduces Rate of Cell Death Induced by Light Exposure

**FIGURE 2:**
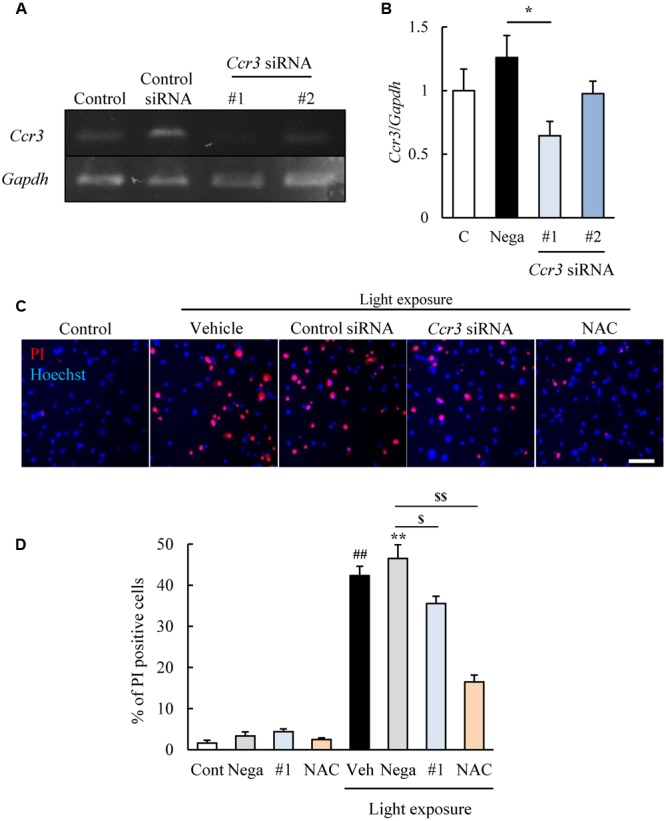
***Ccr3* knockdown suppresses the death of 661W cells induced by light exposure. (A,B)** *Ccr3* knockdown in the cells using *Ccr3* siRNA (#1 and #2). *Ccr3* siRNA #1 decreases the level of the mRNA of *Ccr3* compared to the negative control siRNA, but not #2. **(C,D)** The cell death assay was performed with Hoechst 33342 (blue) and propidium iodide (PI: red). A typical image and quantitative data show that PI-positive cells (dead cells) are increased in the light-exposed group, and *Ccr3* siRNA #1 suppressed the cell death compared to negative control siRNA. *N*-acetylcystein (NAC) was used as a positive control. Data are expressed as means ± SEM (*n* = 3 or 6). ^#, ##^ indicate *P* < 0.05, 0.01 vs. Control (Cont); ^∗, ∗∗^ indicate *P* < 0.05, 0.01 vs. Non-light exposed negative control siRNA (Nega); ^$, $$^ indicate *P* < 0.05, 0.01 vs. Light exposed negative control siRNA (**B**: one-way ANOVA followed by Bonferroni’s test, **D**: one-way ANOVA followed by Tukey’s test). The scale bar represents 50 μm.

We then examined whether CCR3 signaling was activated by light exposure and caused cell death. To do this, we investigated whether inhibition of CCR3 by *Ccr3* knockdown or CCR3 antagonists will reduce the degree of cell death induced by light exposure. First, we tested the *Ccr3* knockdown method on cells of the photoreceptor cell-line with *Ccr3* siRNA #1 and #2. Exposure of 661W cells to *Ccr3* siRNA #1 decreased the level of the mRNA of *Ccr3* by 50% compared to the negative control siRNA. However, siRNA #2 did not alter the level of the mRNA of *Ccr3* (**Figures 2A,B**).

Therefore, we investigated the effect of *Ccr3* siRNA #1 on the light-induced death of cells of the photoreceptor cell line. This cell death assay was performed by double staining of the cells with Hoechst 33342 and PI (Nakanishi et al., 2013). The number of PI-positive cells (dead cells) was increased in the light-exposed group, and exposure to *Ccr3* siRNA #1 significantly reduced the increase in the cell death compared to the negative control siRNA (**Figures 2C,D**). An antioxidant, *N*-acetylcystein (NAC) was used for positive control (Kuse et al., 2014).

**FIGURE 3:**
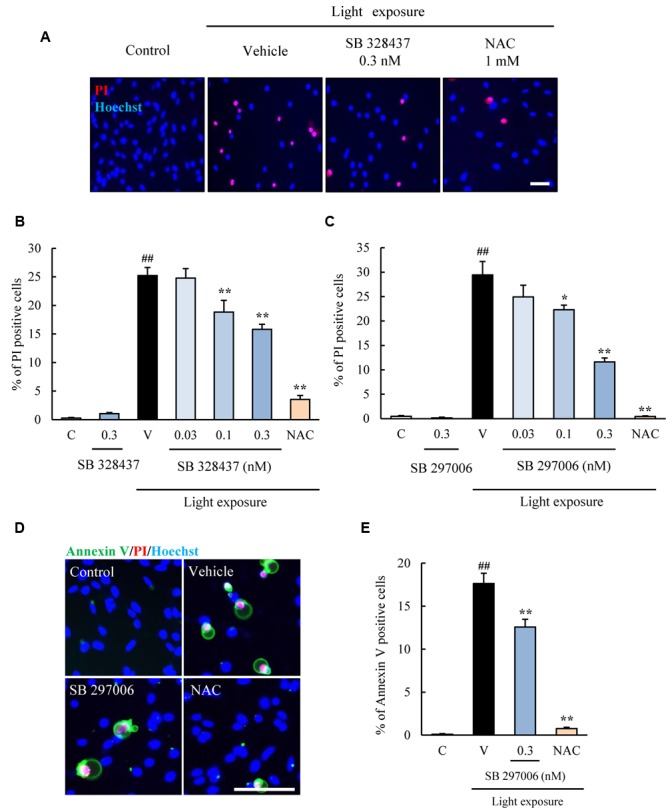
**C-C chemokine receptor type 3 antagonists suppress the death of 661W cells induced by light exposure. (A–C)** Two types of CCR3 antagonists, SB 328437 and SB 297006, were used to examine the role played by CCR3 in the death of 661W cells induced by light exposure. Both CCR3 antagonists reduced the rate of cell death. **(D,E)** The rate of apoptosis was evaluated using Annexin V FITC Apoptosis Detection kit with Annexin V (green), PI (red) and Hoechst 33342 (blue). The rate of annexin V-positive cells was increased in vehicle-treated group by light exposure. SB 297006 and NAC suppressed the apoptosis. *N*-acetylcystein (NAC) was used for a positive control. Data are expressed as mean ± SEM (*n* = 6). ^##^indicates *P* < 0.01 vs. Control (C); ^∗, ∗∗^ indicate *P* < 0.05, 0.01 vs. Vehicle (V) (one-way ANOVA followed by Tukey’s test). The scale bar represents 50 μm.

Next, two types of CCR3 antagonists were used with the same cell death assay. SB 328437 and SB 297006 are selective antagonists of CCR3 (IC_50_ for SB 328437 is 4 nM and for SB 297006 is 2.5 μM). SB 328437 is at least 2500 times more selective for CCR3 than for CXCR1, CXCR2, and CCR7, and SB 297006 is at least 250 times more selective for CCR3 than for CXCR1, CXCR2, CCR1, and CCR7. The two CCR3 antagonists reduced the rate of cell death induced by light exposure significantly (**Figures 3A–C**). Moreover, the rate of apoptosis was evaluated using Annexin V FITC Apoptosis Detection kit. The rate was increased in vehicle-treated group by light exposure. SB 297006 and NAC suppressed the apoptosis (**Figures 3D,E**). These results indicated that the inhibition of CCR3 signaling can reduce the rate of photoreceptor cell death induced by light exposure.

### CCR3 Inhibition Suppresses Production of Reactive Oxygen Species (ROS) and Caspase-3/7 Activation Induced by Light Exposure

**FIGURE 4:**
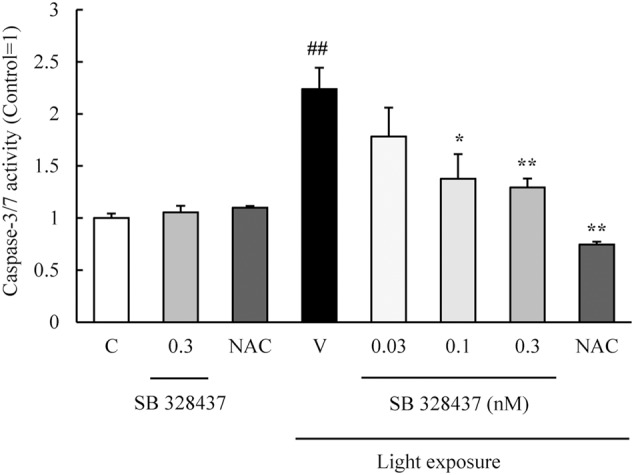
**C-C chemokine receptor type 3 antagonist suppresses the activation of caspase-3/7 induced by light exposure.** The activation of caspase-3/7 was measured with the Caspase-Glo 3/7 Assay. Light exposure activated the caspase-3/7, and a CCR3 antagonist, SB 328437, reduced the activation in a concentration-dependent manner. *N*-acetylcystein (NAC) was used for a positive control. Data are expressed as mean ± SEM (*n* = 4). ^##^indicates *P* < 0.01 vs. Control (C); ^∗, ∗∗^ indicate *P* < 0.05, 0.01 vs. Vehicle (V) (one-way ANOVA followed by Tukey’s test).

**FIGURE 5:**
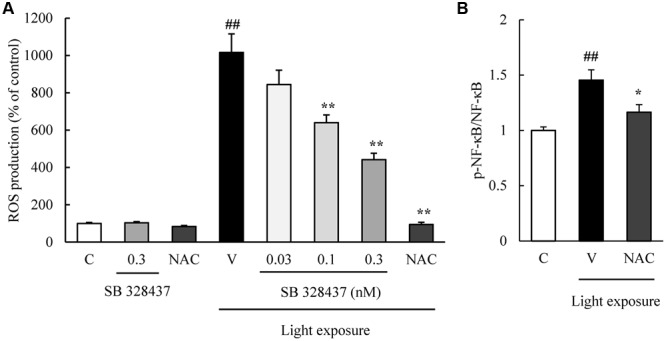
**C-C chemokine receptor type 3 antagonist reduces the degree of reactive oxygen species (ROS) production induced by light exposure. (A)** ROS production was measured by a ROS detecting probe. Light exposure induced the ROS production, and a CCR3 antagonist, SB 328437, reduced the production in a concentration-dependent manner. *N*-acetylcystein (NAC) was used for a positive control. **(B)** NAC treatment suppressed the phosphorylation of NF-κB induced by light exposure. Data are expressed as mean ± SEM (*n* = 4 to 6). ^##^indicates *P* < 0.01 vs. Control (C); ^∗, ∗∗^ indicate *P* < 0.05, 0.01 vs. Vehicle (V) (one-way ANOVA followed by Tukey’s test).

The activation of caspase-3/7 plays a role in the death of the cells of the photoreceptor cell line induced by light exposure (Wu J. et al., 2002). We evaluated the level of activation of caspase-3/7 with the Caspase-Glo 3/7 Assay. Light exposure activated caspase-3/7, and the CCR3 antagonist, SB 328437, suppressed the activation in a concentration-dependent manner (**Figure 4**). Oxidative stress caused by ROS is associated with photoreceptor cell death (Organisciak and Vaughan, 2010). We investigated whether the CCR3 antagonist will reduce the rate of 661W cell death through the inhibition of ROS production using a ROS detecting probe. Light exposure induced the production of ROS, and a CCR3 antagonist suppressed the production in a concentration-dependent manner (**Figure 5A**). Moreover, the decrease of ROS by NAC treatment suppressed the phosphorylation of nuclear factor-kappa B (NF-κB) induced by light exposure (**Figure 5B**). These results indicated that the inhibition of CCR3 reduced the rate of cell death induced by light exposure through the inhibition of ROS generation and activation of caspase-3/7.

## Discussion

Some epidemiological surveys and laboratory studies have shown that apolipoprotein E (Souied et al., 1998; Levy et al., 2015), superoxide dismutase (Kimura et al., 2000), complement factor H (Edwards et al., 2005; Haines et al., 2005), and CX3C chemokine receptor 1 (CX3CR1) (Tuo et al., 2004; Combadière et al., 2007) are key factors in the development of dry AMD. CX3CR1 and CCR2 are the receptors for CX3C chemokine ligand 1 (CX3CL1) and these two chemokine receptors have been detected in the subretinal space of eyes with dry AMD (Combadière et al., 2007; Sennlaub et al., 2013). Thus, an inhibition of CCL2/CCR2 has the potential of reducing the degree of the chronic inflammation in experimental dry AMD. However, it remains unknown how CCR3 signaling is associated with even experimental dry AMD model.

C-C chemokine receptor type 3 is expressed in normal murine retinas (Wang et al., 2016) and our western blotting results showed the expression of CCR3 in 661W cells, a mouse photoreceptor-derived cell line. We investigated the association of CCR3 with the light-induced death of 661W cells which are cells of a photoreceptor cell line that express many cone-specific antigens. These cells have been used as an *in vitro* model of dry AMD (Krishnamoorthy et al., 1999; Kanan et al., 2007). Our results showed that CCR3 was expressed in the 661W cells, and light exposure up-regulated the activity of CCR3 which will then enhance the death of the 661W cells (**Figure 1**). We also found that inhibition of CCR3 depressed the rate of death of the 661W cells that was induced by light exposure. In particular, our results showed that an inhibition of CCR3 by *Ccr3* knockdown or by CCR3 antagonists, SB 328437 and SB 297006, decreased the rate of death of 661W cells induced by light exposure (**Figures 2, 3**). Both CCR3 antagonists depressed the cell death more strongly than *Ccr3* knockdown. This was due to the weak efficacy of the mRNA in the *Ccr3* knockdown (by 50% decrease) although *Ccr3* siRNA #1 successfully decreased the level of the mRNA of *Ccr3* (**Figures 2A,B**). Moreover, a difference in the degree of decrease in the death of 661W cells was observed between the two CCR3 antagonists (**Figure 3**). It has been reported that SB 297006 was the potent inhibitor of CCR1 (Sabroe et al., 2000), and our results showed that SB 297006 reduced the rate of the death of 661W cells more strongly than SB 328437 although the IC_50_ of SB 328437 (4 nM) is lower than that of SB 297006 (2.5 μM). It is interesting to note that the activation of CCR1 may be associated with the photoreceptor degeneration in rd mice (Zeng et al., 2011). Thus, SB 297006 might have strong protective effects on 661W cells through the inhibition of CCR1 signaling as well as through CCR3 signaling. The suppression of both CCR3 and CCR1 signaling may have the stronger potential than the suppression of selective CCR3 signaling against the photoreceptor degeneration which is observed in dry AMD and retinal pigmentosa. While, CCR3 inhibitor plays a protective role in injured primary cortical cultures under oxygen glucose deprivation (Zhang et al., 2016). Therefore, the suppression of CCR3 signaling may be one of therapeutic candidates for the loss of neurons.

The results of an earlier study suggested that ROS increase the expression of CCR3 in choroidal endothelial cells (Wang et al., 2011). Light exposure induced the production of ROS in 661W cells and caused apoptosis (Krishnamoorthy et al., 1999) probably through the cone opsins but not the rhodopsin (Tan et al., 2004). In addition, light exposure has been shown to increase the mRNA level of tumor necrosis factor-α (TNF-α) and NF-κB (Wu T. et al., 2002; Yang et al., 2007). These findings are relevant because TNF-α induces an up-regulation of CCR3, and the level of expression of CCR3 is reduced by inhibiting the IκB kinase-β activity and NF-κB activation in human dermal fibroblasts (Huber et al., 2002; Lee et al., 2006). In this study, the phosphorylation of NF-κB was suppressed by decreasing ROS level with treatment of NAC (**Figure 5B**). Thus, ROS and subsequent TNF-α and NF-κB may induce the death of 661W cells through the expression of CCR3 although the downstream CCR3-associated responses of the death signaling pathway has not been determined. More investigations are required to identify the CCR3-associated responses on light-exposed 661W cells.

## Conclusion

The results showed that CCR3 plays a role in the death of 661W cells that is induced by light exposure, and inhibition of CCR3 will reduce the rate of death of the 661W cells. Because CCR3 is expressed in endothelial cells in the CNV membranes of eyes with dry AMD and has been used as an *in vitro* model of dry AMD, these findings suggest that CCR3 inhibition should be considered as a therapy for not only dry but also wet AMD through its protective effects on photoreceptor cells and the suppression of CNV. However, it still needs to be determined whether CCR3 inhibition is safe (Takeda et al., 2009; Wang et al., 2016). More investigations are required to determine the effects of CCR3 inhibition on the retina in normal or pathological conditions.

## Author Contributions

YKu, KT, YKa, MS, and HH conceived and designed the experiments. YKu and YKa performed the analysis and the experiments. YKu, KT, and HH wrote the paper. All authors reviewed the manuscript.

## Conflict of Interest Statement

The authors declare that the research was conducted in the absence of any commercial or financial relationships that could be construed as a potential conflict of interest.

## References

[B1] Ambati J. Ambati B. K. Yoo S. H. Ianchulev S. Adamis A. P. (2003). Age-related macular degeneration: etiology, pathogenesis, and therapeutic strategies. *Surv. Ophthalmol.* 48 257–293. 10.1016/S0039-6257(03)00030-4 12745003

[B2] Beatty S. Koh H. Phil M. Henson D. Boulton M. (2000). The role of oxidative stress in the pathogenesis of age-related macular degeneration. *Surv. Ophthalmol.* 45 115–134. 10.1016/S0039-6257(00)00140-5 11033038

[B3] Combadière C. Feumi C. Raoul W. Keller N. Rodéro M. Pézard A. (2007). CX3CR1-dependent subretinal microglia cell accumulation is associated with cardinal features of age-related macular degeneration. *J. Clin. Invest.* 117 2920–2928. 10.1172/JCI31692 17909628 PMC1994614

[B4] Dunaief J. L. Dentchev T. Ying G.-S. Milam A. H. (2002). The role of apoptosis in age-related macular degeneration. *Arch. Ophthalmol.* 120 1435–1442. 10.1001/archopht.120.11.1435 12427055

[B5] Edwards A. O. Ritter R. Abel K. J. Manning A. Panhuysen C. Farrer L. A. (2005). Complement factor H polymorphism and age-related macular degeneration. *Science* 308 421–424. 10.1126/science.1110189 15761121

[B6] Haines J. L. Hauser M. A. Schmidt S. Scott W. K. Olson L. M. Gallins P. (2005). Complement factor H variant increases the risk of age-related macular degeneration. *Science* 308 419–421. 10.1126/science.1110359 15761120

[B7] Huber M. A. Denk A. Peter R. U. Weber L. Kraut N. Wirth T. (2002). The IKK-2/Ikappa Balpha /NF-kappa B pathway plays a key role in the regulation of CCR3 and eotaxin-1 in fibroblasts. A critical link to dermatitis in Ikappa Balpha-deficient mice. *J. Biol. Chem.* 277 1268–1275. 10.1074/jbc.M109358200 11694538

[B8] Kanan Y. Moiseyev G. Agarwal N. Ma J.-X. Al-Ubaidi M. R. (2007). Light induces programmed cell death by activating multiple independent proteases in a cone photoreceptor cell line. *Invest. Ophthalmol. Vis. Sci.* 48 40–51. 10.1167/iovs.06-0592 17197514

[B9] Kimura K. Isashiki Y. Sonoda S. Kakiuchi-Matsumoto T. Ohba N. (2000). Genetic association of manganese superoxide dismutase with exudative age-related macular degeneration. *Am. J. Ophthalmol.* 130 769–773. 10.1016/S0002-9394(00)00552-3 11124296

[B10] Krishnamoorthy R. R. Crawford M. J. Chaturvedi M. M. Jain S. K. Aggarwal B. B. Al-Ubaidi M. R. (1999). Photo-oxidative stress down-modulates the activity of nuclear factor-kappaB via involvement of caspase-1, leading to apoptosis of photoreceptor cells. *J. Biol. Chem.* 274 3734–3743. 10.1074/jbc.274.6.3734 9920926

[B11] Kuse Y. Ogawa K. Tsuruma K. Shimazawa M. Hara H. (2014). Damage of photoreceptor-derived cells in culture induced by light emitting diode-derived blue light. *Sci. Rep.* 4:5223 10.1038/srep05223 PMC4048889 24909301

[B12] Lee J. Jung E. Kim Y. Lee J. Park J. Hong S. (2006). Rosmarinic acid as a downstream inhibitor of IKK-beta in TNF-alpha-induced upregulation of CCL11 and CCR3. *Br. J. Pharmacol.* 148 366–375. 10.1038/sj.bjp.0706728 16604092 PMC1751564

[B13] Levy O. Calippe B. Lavalette S. Hu S. J. Raoul W. Dominguez E. (2015). Apolipoprotein E promotes subretinal mononuclear phagocyte survival and chronic inflammation in age-related macular degeneration. *EMBO Mol. Med.* 7 211–226. 10.15252/emmm.201404524 25604058 PMC4328649

[B14] Liang F.-Q. Godley B. F. (2003). Oxidative stress-induced mitochondrial DNA damage in human retinal pigment epithelial cells: a possible mechanism for RPE aging and age-related macular degeneration. *Exp. Eye Res.* 76 397–403. 12634104 10.1016/s0014-4835(03)00023-x

[B15] Nakanishi T. Shimazawa M. Sugitani S. Kudo T. Imai S. Inokuchi Y. (2013). Role of endoplasmic reticulum stress in light-induced photoreceptor degeneration in mice. *J. Neurochem.* 125 111–124. 10.1111/jnc.12116 23216380

[B16] Organisciak D. T. Vaughan D. K. (2010). Retinal light damage: mechanisms and protection. *Prog. Retin. Eye Res.* 29 113–134. 10.1016/j.preteyeres.2009.11.004 19951742 PMC2831109

[B17] Sabroe I. Peck M. J. Van Keulen B. J. Jorritsma A. Simmons G. Clapham P. R. (2000). A small molecule antagonist of chemokine receptors CCR1 and CCR3. Potent inhibition of eosinophil function and CCR3-mediated HIV-1 entry. *J. Biol. Chem.* 275 25985–25992. 10.1074/jbc.M908864199 10854442

[B18] Sennlaub F. Auvynet C. Calippe B. Lavalette S. Poupel L. Hu S. J. (2013). CCR2(+) monocytes infiltrate atrophic lesions in age-related macular disease and mediate photoreceptor degeneration in experimental subretinal inflammation in *Cx3cr1* deficient mice. *EMBO Mol. Med.* 5 1775–1793. 10.1002/emmm.201302692 24142887 PMC3840491

[B19] Shahinfar S. Edward D. P. Tso M. O. (1991). A pathologic study of photoreceptor cell death in retinal photic injury. *Curr. Eye Res.* 10 47–59. 10.3109/02713689109007610 2029848

[B20] Souied E. H. Benlian P. Amouyel P. Feingold J. Lagarde J. P. Munnich A. (1998). The epsilon4 allele of the apolipoprotein E gene as a potential protective factor for exudative age-related macular degeneration. *Am. J. Ophthalmol.* 125 353–359. 10.1016/S0002-9394(99)80146-9 9512153

[B21] Takeda A. Baffi J. Z. Kleinman M. E. Cho W. G. Nozaki M. Yamada K. (2009). CCR3 is a target for age-related macular degeneration diagnosis and therapy. *Nature* 460 225–230. 10.1038/nature08151 19525930 PMC2712122

[B22] Tan E. Ding X.-Q. Saadi A. Agarwal N. Naash M. I. Al-Ubaidi M. R. (2004). Expression of cone-photoreceptor-specific antigens in a cell line derived from retinal tumors in transgenic mice. *Invest. Ophthalmol. Vis. Sci.* 45 764–768. 10.1167/iovs.03-1114 14985288 PMC2937568

[B23] Tuo J. Smith B. C. Bojanowski C. M. Meleth A. D. Gery I. Csaky K. G. (2004). The involvement of sequence variation and expression of CX3CR1 in the pathogenesis of age-related macular degeneration. *FASEB J.* 18 1297–1299. 10.1096/fj.04-1862fje 15208270 PMC1971128

[B24] Wang H. Han X. Gambhir D. Becker S. Kunz E. Liu A. J. (2016). Retinal inhibition of CCR3 induces retinal cell death in a murine model of choroidal neovascularization. *PLoS ONE* 11:e0157748 10.1371/journal.pone.0157748 PMC4911089 27309355

[B25] Wang H. Wittchen E. S. Jiang Y. Ambati B. Grossniklaus H. E. Elizabeth Hartnett M. (2011). Upregulation of CCR3 by age-related stresses promotes choroidal endothelial cell migration via VEGF-dependent and -independent signaling. *Investig. Ophthalmol. Vis. Sci.* 52 8271–8277. 10.1167/iovs.11-8230 21917937 PMC3208059

[B26] Wu J. Gorman A. Zhou X. Sandra C. Chen E. (2002). Involvement of caspase-3 in photoreceptor cell apoptosis induced by in vivo blue light exposure. *Invest. Ophthalmol. Vis. Sci.* 43 3349–3354. 12356844

[B27] Wu T. Chen Y. Chiang S. K. Tso M. O. (2002). NF-kappaB activation in light-induced retinal degeneration in a mouse model. *Invest. Ophthalmol. Vis. Sci.* 43 2834–2840. 12202499

[B28] Yang L.-P. Zhu X.-A. Tso M. O. M. (2007). Role of NF-kappaB and MAPKs in light-induced photoreceptor apoptosis. *Invest. Ophthalmol. Vis. Sci.* 48 4766–4776. 10.1167/iovs.06-0871 17898303

[B29] Zeng H. Lu Q. Liu Q. Liu K.-G. Wang N. (2011). The role of CCR1 expression in the retinal degeneration in rd mice. *Curr. Eye Res.* 36 264–269. 10.3109/02713683.2010.535133 21275605

[B30] Zhang J. Wang H. Sherbini O. Ling-Lin Pai E. Kang S.-U. Kwon J.-S. (2016). High-content genome-wide RNAi screen reveals CCR3 as a key mediator of neuronal cell death. *eNeuro* 3:e0185-16 10.1523/ENEURO.0185-16.2016 PMC5075945 27822494

